# Retraction: miR-34a modulates Angiotensin II-induced myocardial hypertrophy by direct inhibition of ATG9A expression and Autophagic activity

**DOI:** 10.1371/journal.pone.0319360

**Published:** 2025-02-19

**Authors:** The PLOS One Editors

After this article [1] was published, concerns were raised regarding results presented in Figs 1, and 3-6.

Specifically:

- The following lanes appear similar despite representing different experimental conditions:
  ◦ Fig 3D ATG9A lane 1 and Fig 5D ATG9A lane 3
  ◦ Fig 4D GAPDH lane 2 and Fig 4D GAPDH lane 3 flipped horizontally
  ◦ Fig 4D GAPDH lane 1 and Fig 5C GAPDH lane 2
- In the Fig 5C Con AngII p62 panel there appears to be a vertical discontinuity in the background between lanes 1 and 2.
- The Fig 1B Sham and TAAC panels of [1] appear similar to Figs 7A and 7E of [2] respectively.
- The following results appear similar despite representing different experimental conditions:
  ◦ Fig 4D GAPDH panel of [1] and Fig 3 GAPDH panel of [3].
  ◦ Fig 5D ATG9A panel of [1] and Fig 3 ATG9a panel of [3].
  ◦ Fig 5D LC3 panel of [1] and Fig 3 LC3 panel of [3].
  ◦ Fig 6E Ang II+NC panel of [1] and Fig 4 Sham panel of [3].

The authors did not respond to editorial requests for a response and underlying data. In light of the above unresolved concerns that question the integrity and reliability of the reported results and conclusions, the *PLOS One* Editors retract this article.

All authors either did not respond directly or could not be reached.
